# Supplementary material for: Computational Design of Novel Cyclic Peptides Endowed with Autophagy-Inhibiting Activity on Cancer Cell Lines
Source: Int J Mol Sci. 2024 Apr 24;25(9):4622. doi: 10.3390/ijms25094622 (PMC11083565; doi:10.3390/ijms25094622)
Supplement: Supplementary file 1 [file ijms-25-04622-s001.zip › ijms-2958988-supplementary.pdf]

Supplementary Materials

Computational Design of Novel Cyclic Peptides Endowed with Autophagy Inhibiting Activity on Cancer Cell Lines

Marco Albani[1], Enrico Mario Alessandro Fassi[1,*], Roberta Manuela Moretti[2], Mariangela Garofalo[3], Marina Montagnani Marelli[2], Gabriella Roda[1], Jacopo Sgrignani[4], Andrea Cavalli[4,5], and Giovanni Grazioso[1,*]

[1] Department of Pharmaceutical Sciences, Università degli Studi di Milano, Via L. Mangiagalli 25, 20133 Milano, Italy;

[2] Department of Pharmacological and Biomolecular Sciences, Università degli Studi di Milano, Via Balzaretti 9, 20133 Milano, Italy;

[3] Department of Pharmaceutical and Pharmacological Sciences, Università di Padova, Via F. Marzolo 5, 35131 Padova, Italy;

[4] Institute for Research in Biomedicine (IRB), Via Chiesa 5, 6500 Bellinzona, Switzerland;

[5] Swiss Institute of Bioinformatics (SIB), University of Lausanne, Quartier UNIL-Sorge, Bâtiment Amphipôle, 1015 Lausanne, Switzerland.

* Correspondence: enrico.fassi@unimi.it, giovanni.grazioso@unimi.it

Table S1. Computational alanine scanning data on LIR2-RavZ peptide.

**Mutation on LIR2-RavZ**	**ΔAffinity [kcal/mol]**
DIDEFDLLEGDE	0.0
1(ASP->ALA)	-0.8
2(ILE->ALA)	+1.4
3(ASP->ALA)	+13.1
4(GLU->ALA)	+17.2
5(PHE->ALA)	+19.7
6(ASP->ALA)	+6.6
7(LEU->ALA)	+5.0
8(LEU->ALA)	+3.8
9(GLU->ALA)	+4.3
11(ASP->ALA)	+7.3
12(GLU->ALA)	+1.2

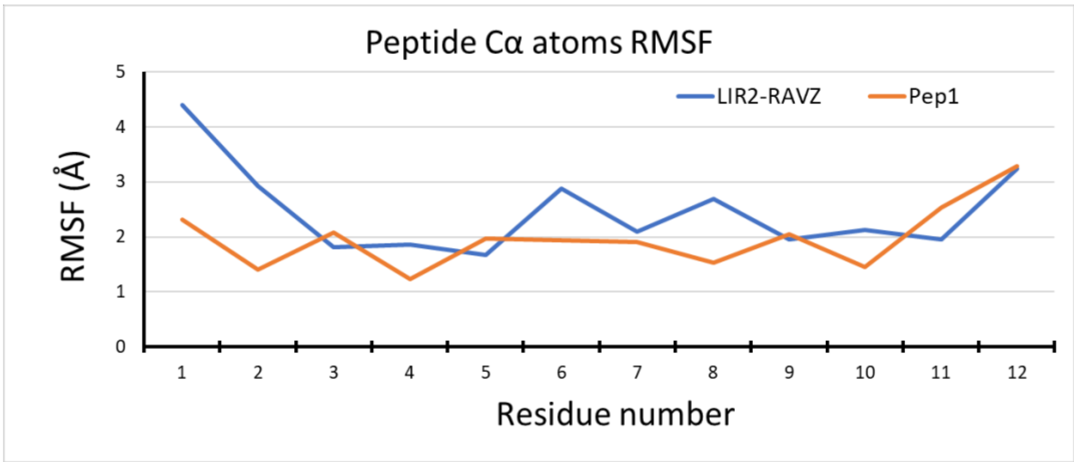

Figure S1. Cα atoms RMSF over the MD simulation time (250 ns).

Table S2. Computational alanine scanning data on Pep2. Residues in positions 10 and 12 were not mutated since they are needed to constrain the peptide.

**Mutation on Pep2**	**ΔAffinity [kcal/mol]**
Ac-DIDEFDLLECDC-*NH*$_2$	0.0
1(ASP->ALA)	+1.3
2(ILE->ALA)	+5.8
3(ASP->ALA)	+5.8
4(GLU->ALA)	+11.2
5(PHE->ALA)	+19.9
6(ASP->ALA)	+1.9
7(LEU->ALA)	+16.5
8(LEU->ALA)	+7.3
9(GLU->ALA)	+9.2
11(ASP->ALA)	+7.5

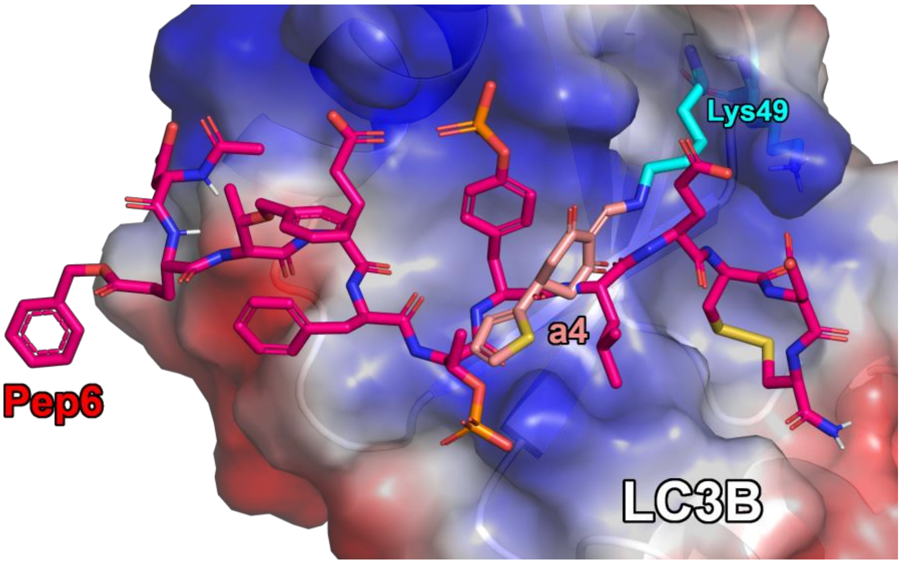

Figure S2. Superimposition of LC3B/Pep6 complex and LC3B/a4 X-ray structure (PDB code 7ELG). The protein surface is colored depending on the atomic partial charges of the protein residues: blue for positive and red for negative charges, respectively. Pep6 and the covalent ligand a4 are shown as magenta and pink sticks, respectively, while both the LC3B-Lys49 of LC3B/Pep6 complex and LC3B/a4 X-ray structure are represented as cyan sticks.

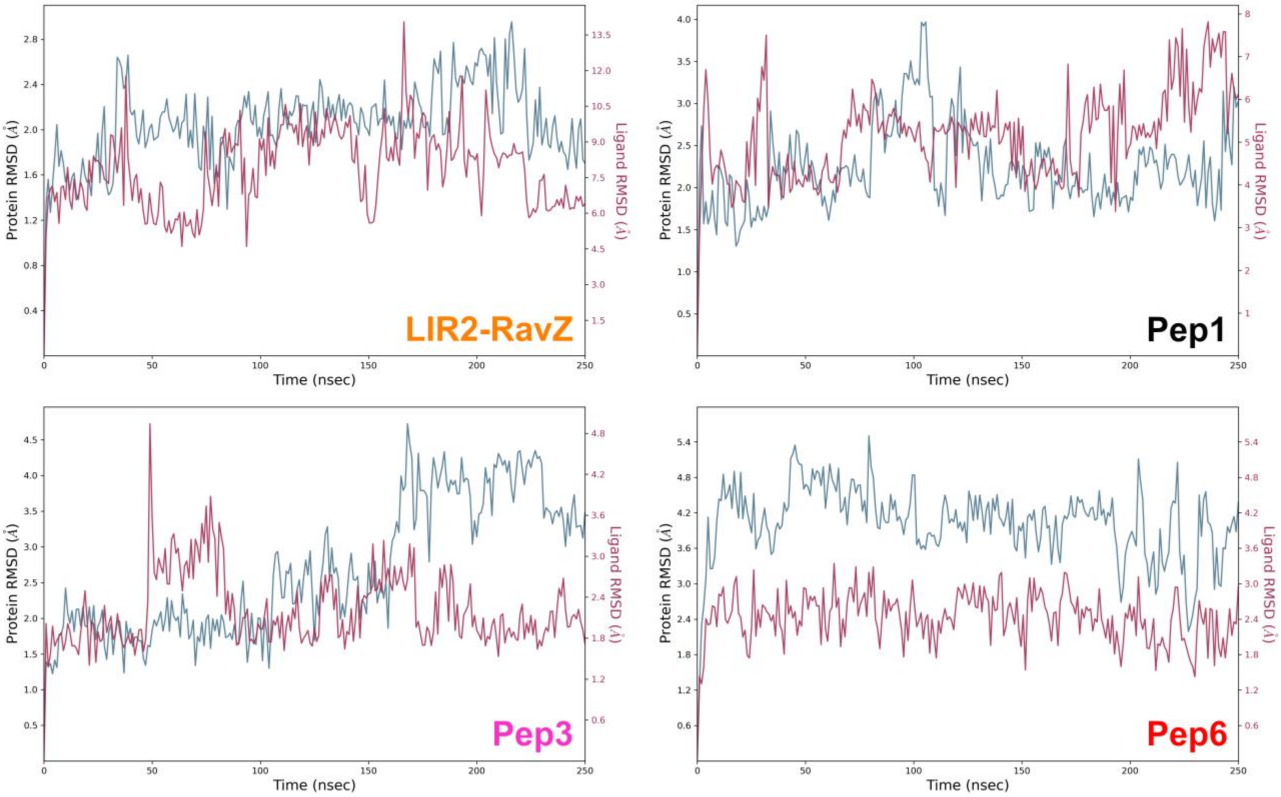

Figure S3. Cα atoms RMSD plots of the main simulated systems over 250 ns of MD simulation time.

Table S3. Dataset overview of the Microscale Thermophoresis (MST) experiments accomplished using a fixed concentration of human recombinant His-tagged LC3B protein (10 nM) and different concentrations of LIR2-RavZ peptide, used as positive control, and Pep8. Two independent experiments were accomplished to compute the K_d value.

Peptide	MST Power	Exc. Power	Temp.	[Ligand] Range	Time	RA	SNR	K_d (nM)
LIR2-RavZ	40%	20%	25 °C	15.6 µM – 0.477 nM	5 s	4.4	9.1	428 ± 162
Pep6	40%	20%	25 °C	31.3 µM – 0.954 nM	10 s	5.1	9.2	159 ± 56

RA = Response Amplitude; SNR = Signal-to-Noise Ratio.

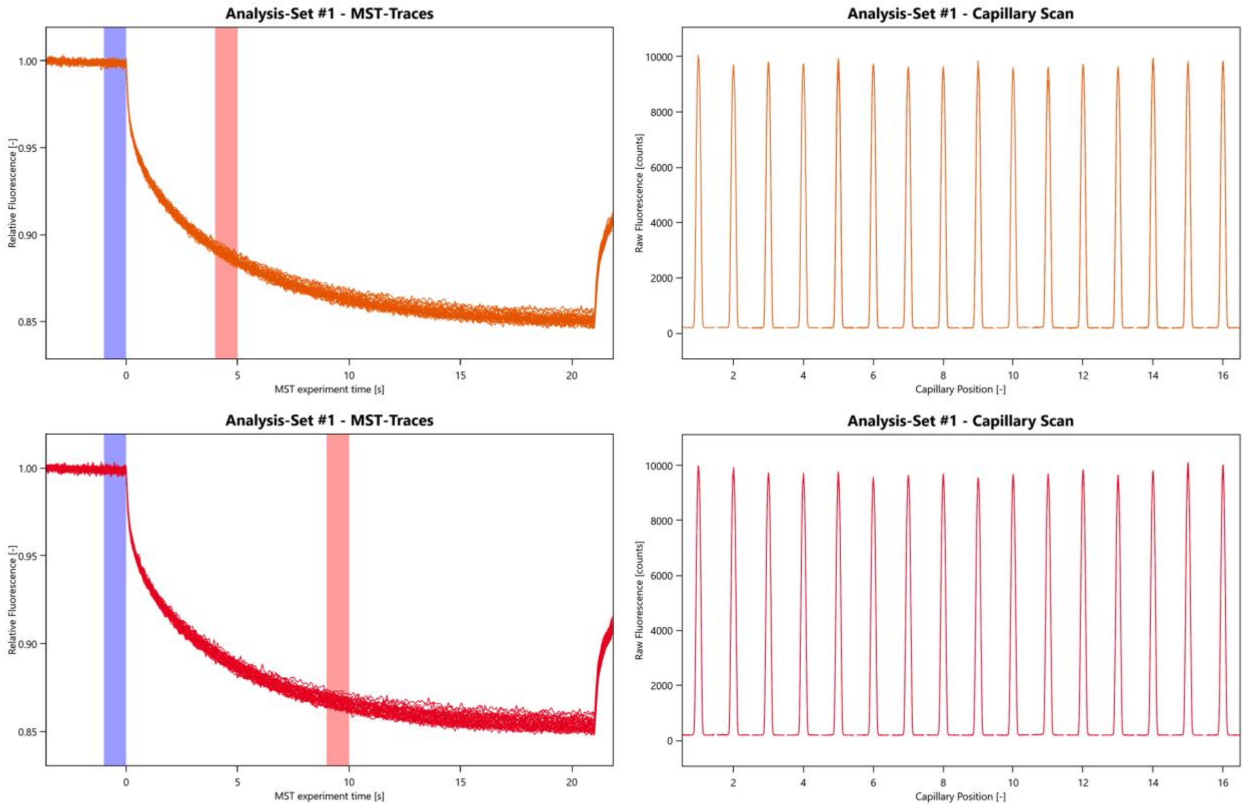

Figure S4. MST traces and raw fluorescence values, of each capillary scanned, acquired in the MST experiments accomplished to evaluate the binding of peptide LIR2-RavZ (top, orange) and Pep6 (bottom, red) to human recombinant LC3B protein.

HPLC Analysis

Name	:LIR2-RAVZ
Sequence	:DIDEFDLLEGDE(desalted)
Lot.No	:PCM15546-1-0919
Pump A	:0.1%Trifluoroacetic in 100% water
Pump B	:0.1%Trifluoroacetic in 100% acetonitrile
Total Flow	:1ml/min
Wavelength	:220nm
Analytical column type	:SHIMADZU Inertsil ODS-SP(4.6*250mm*5um)
Dissolution method	:100%H2O
Inj. Volume	:14 uL

Time	Module	Action	Value
0.01	Pumps	B.Conc	10
20.00	Pumps	B.Conc	50
23.00	Pumps	B.Conc	100
38.00	Pumps	B.Conc	100
40.00	Pumps	B.Conc	10
50.00	Controller	Stop	

Chromatogram

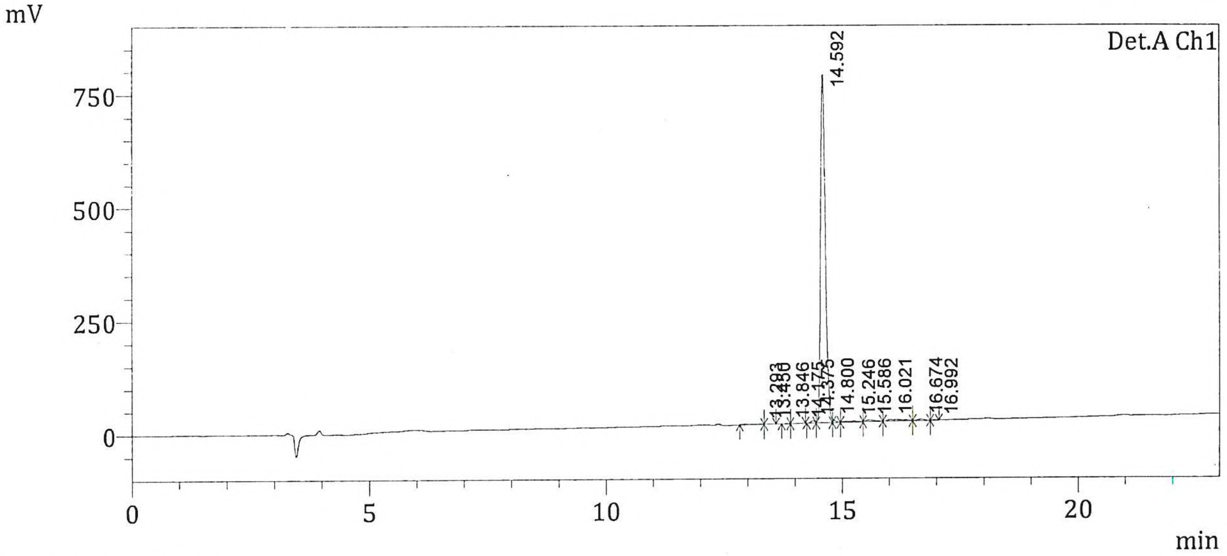

1 Det.A Ch1/220nm

PeakTable

Detector A Ch1 220nm

Peak#	Ret. Time	Area	Height	Area %	Height %
1	13.293	10034	503	0.177	0.063
2	13.450	3978	373	0.070	0.047
3	13.846	2454	361	0.043	0.045
4	14.175	2442	140	0.043	0.018
5	14.375	30812	4991	0.545	0.627
6	14.592	5382062	769760	95.173	96.637
7	14.800	29482	6576	0.521	0.825
8	15.246	37612	2418	0.665	0.304
9	15.586	44500	3352	0.787	0.421
10	16.021	78804	3973	1.394	0.499
11	16.674	31222	3782	0.552	0.475
12	16.992	1654	322	0.029	0.040
Total		5655055	796550	100.000	100.000

Pepmic Co., Ltd

Tel: +86-512-65834896 Email: info@pepmic.com Web: www.pepmic.com

Address: 12 Datong Road, High-tech Development Zone, Suzhou, China 215151

Pepmic Co., Ltd
Tel: +86-512-65834896
Email: info@pepmic.com
Web: www.pepmic.com
Address: 12 Datong Road, High-tech Development Zone, Suzhou, China 215151

pepmic COMMITTED TO LIFE SCIENCE

MS Spectrum Graph

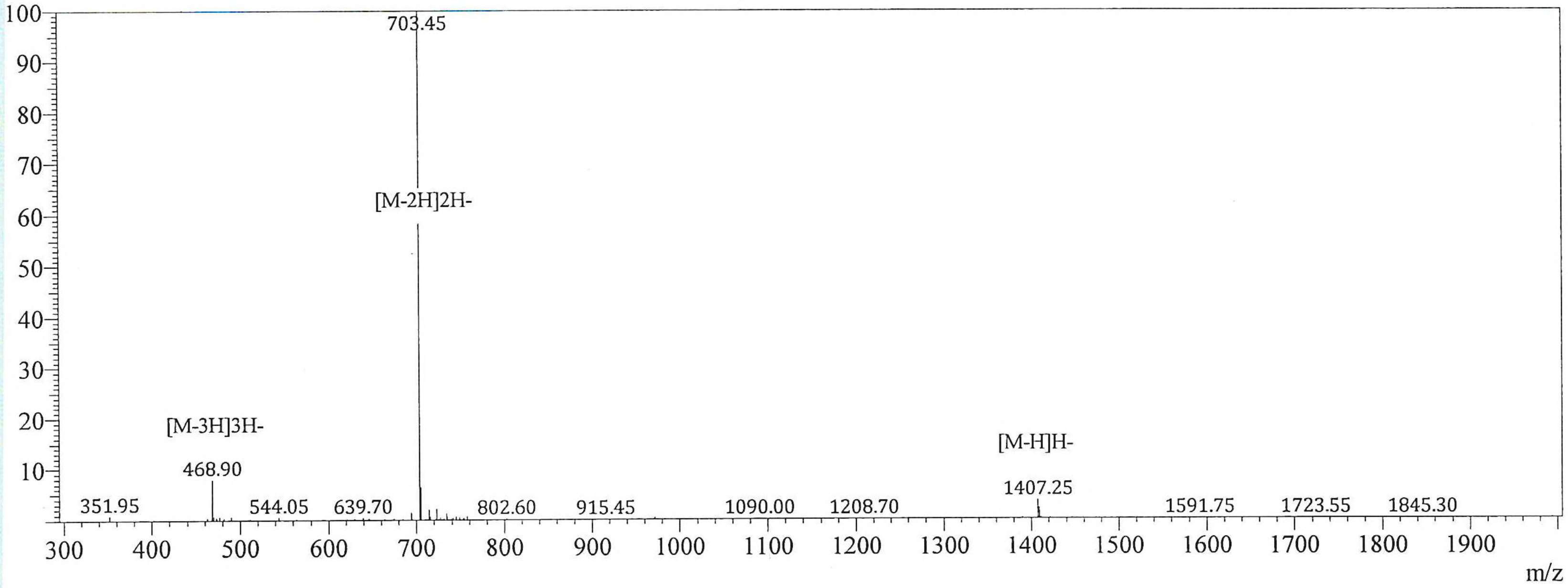

Sample Information

Dissolution method	:5%NH3.H2O+8%ACN+87%H2O	Interface	:ESI	Prerod Bias	:+1.5kv
Modified Date	:2022/10/11	Nebulizing Gas Flow	:1.50L/min	Detector	:-0.2kv
Injection Volume	:1ul	CDL Temp	:250C	T.Flow	:0.2ml/min
Heat Block Temp	:200	CDL Volt	:0v	B.conc	:50%H2O/50%MEOH

Name	:LIR2-RAVZ
Sequence	:DIDEFDLLEGDE(desalted)
Lot.No	:PCM15546-1-0919
Theoretical	:1409.39
Observed	:1408.90

COMMITTED TO LIFE SCIENCE

HPLC Analysis

Name :Pep3
Sequence :Ac-D{E(Bz)}{T(Bz)}EF(pT)(pY)LEC*DC*-NH2(desalt)
Lot.No :PCM15768-0719
Pump A :0.1%Trifluoroacetic in 100% water
Pump B :0.1%Trifluoroacetic in 100% acetonitrile
Total Flow :1ml/min
Wavelength :220nm
Analytical column type :SHIMADZU Inertsil ODS-SP(4.6*250mm*5um)
Dissolution method :15%ACN+85%H2O
Inj. Volume :13 uL

Time	Module	Action	Value
0.01	Pumps	B.Conc	30
20.00	Pumps	B.Conc	70
23.00	Pumps	B.Conc	100
38.00	Pumps	B.Conc	100
40.00	Pumps	B.Conc	30
50.00	Controller	Stop	

Chromatogram

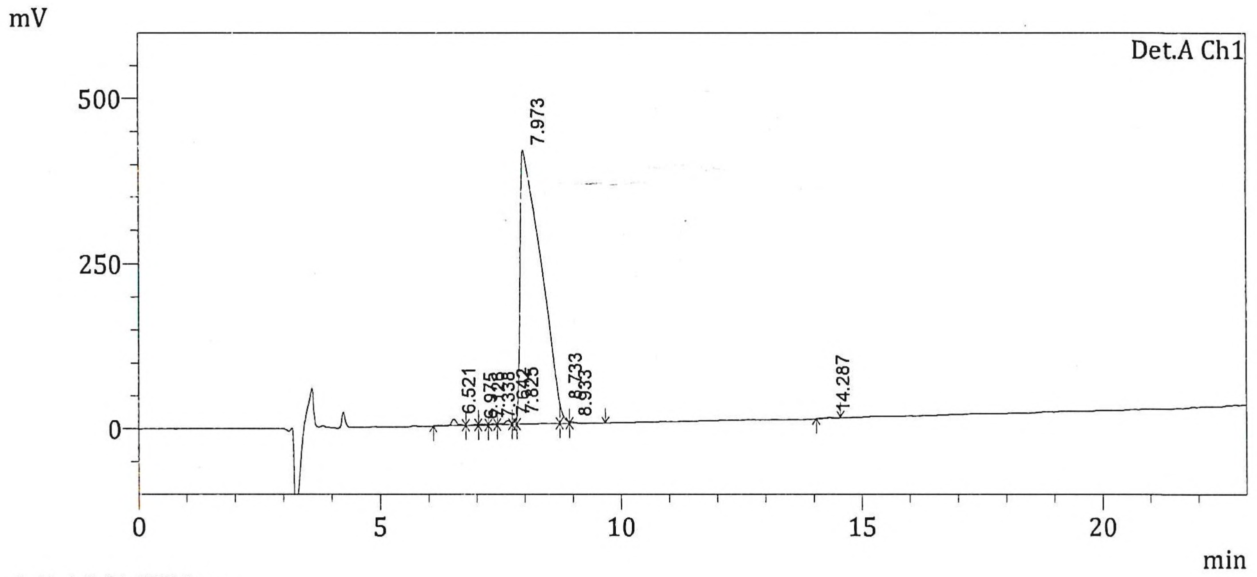

1 Det.A Ch1/220nm

PeakTable

Detector A Ch1 220nm

Peak#	Ret. Time	Area	Height	Area %	Height %
1	6.521	89432	10327	0.695	2.155
2	6.975	15351	1773	0.119	0.370
3	7.126	23245	2385	0.181	0.497
4	7.338	21389	2453	0.166	0.512
5	7.642	60719	6411	0.472	1.338
6	7.825	30795	8618	0.239	1.798
7	7.973	12471083	414702	96.851	86.519
8	8.733	131096	28868	1.018	6.023
9	8.933	20294	2842	0.158	0.593
10	14.287	13185	941	0.102	0.196
Total		12876589	479319	100.000	100.000

Pepmic Co., Ltd
Tel: +86-512-65834896 Email: info@pepmic.com Web: www.pepmic.com
Address: 12 Datong Road, High-tech Development Zone, Suzhou, China 215151

pepmic
COMMITTED TO LIFE SCIENCE

MS Spectrum Graph

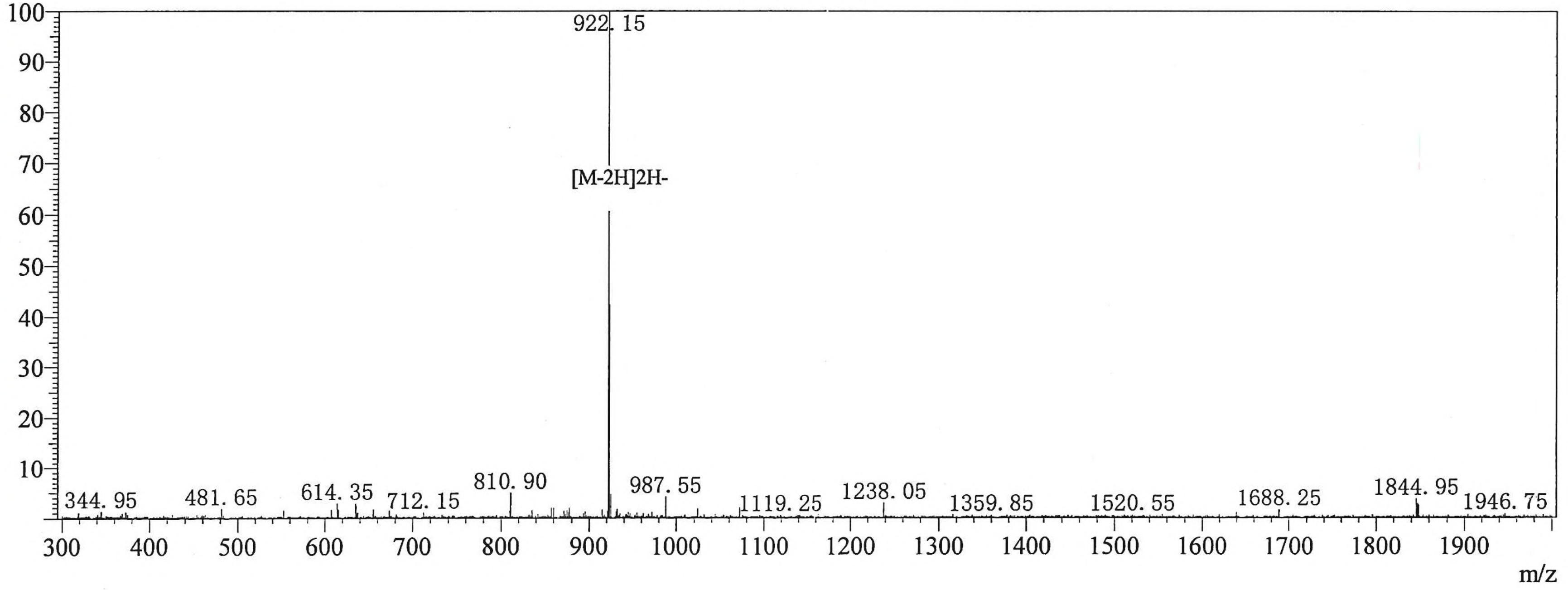

Sample Information

Dissolution method	:5%NH3.H2O+8%ACN+87%H2O	Interface	:ESI	Prerod Bias	:+1.5kv
Modified Date	:2023/09/22	Nebulizing Gas Flow	:1.50L/min	Detector	:-0.2kv
Injection Volume	:1ul	CDL Temp	:250C	T.Flow	:0.2ml/min
Heat Block Temp	:200	CDL Volt	:0v	B.conc	:50%H2O/50%MEOH

Name	:Pep3
Sequence	:Ac-D{E(Bz)}{T(Bz)}EF(pT)(pY)LEC*DC*-NH2(desalt)
Lot.No	:PCM15768-0719
Theoretical	:1846.70
Observed	:1846.30

Pepmic Co., Ltd
Tel: +86-512-65834896 Email: info@pepmic.com Web: www.pepmic.com
Address: 12 Datong Road, High-tech Development Zone, Suzhou, China 215151

HPLC Analysis

Name	:Pep6
Sequence	:Ac-D{E(Bn)}{t(Bn)}Ef(pt)(pY)LEc*Dc*-NH2(desalted)
Lot.No	:PCM15546-3-0919
Pump A	:0.1%Trifluoroacetic in 100% water
Pump B	:0.1%Trifluoroacetic in 100% acetonitrile
Total Flow	:1ml/min
Wavelength	:220nm
Analytical column type	:SHIMADZU Inertsil ODS-SP(4.6*250mm*5um)
Dissolution method	:15%ACN+85%H2O
Inj. Volume	:12 uL

Time	Module	Action	Value
0.01	Pumps	B.Conc	25
20.00	Pumps	B.Conc	65
23.00	Pumps	B.Conc	100
38.00	Pumps	B.Conc	100
40.00	Pumps	B.Conc	25
50.00	Controller	Stop	

Chromatogram

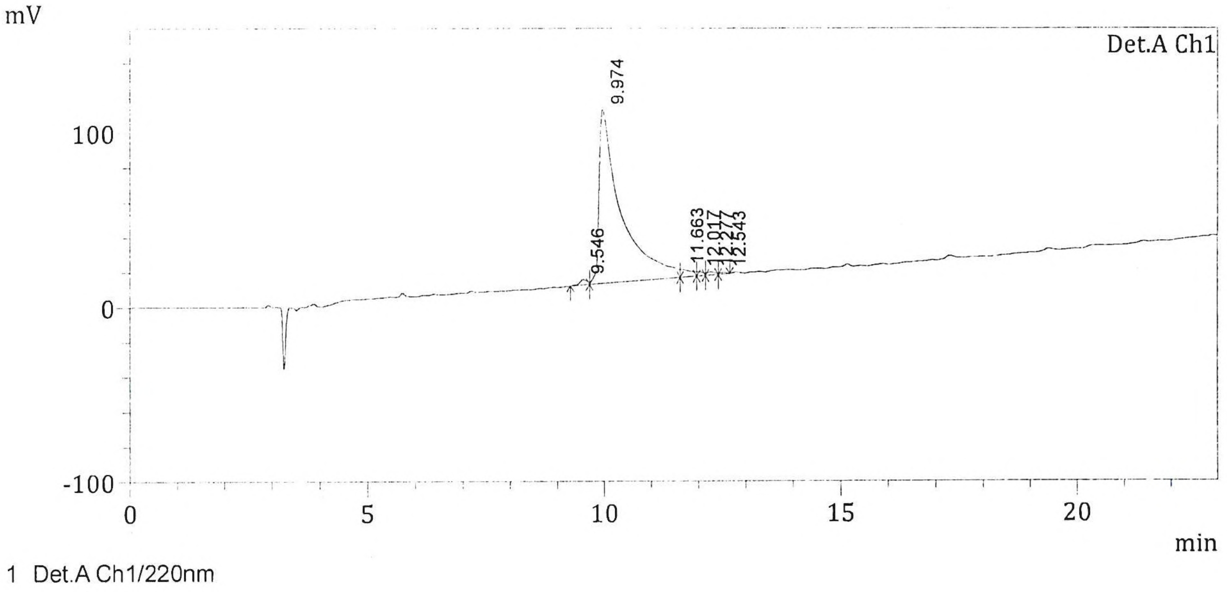

1 Det.A Ch1/220nm

PeakTable

Detector A Ch1 220nm

Peak#	Ret. Time	Area	Height	Area %	Height %
1	9.546	35947	3277	1.052	2.903
2	9.974	3253816	99647	95.194	88.284
3	11.663	72093	4546	2.109	4.027
4	12.017	24073	2544	0.704	2.254
5	12.277	22670	1874	0.663	1.661
6	12.543	9490	983	0.278	0.871
Total		3418089	112870	100.000	100.000

Pepmic Co., Ltd

Tel: +86-512-65834896 Email: info@pepmic.com Web: www.pepmic.com

Address: 12 Datong Road, High-tech Development Zone, Suzhou, China 215151

Pepmic Co., Ltd
Tel: +86-512-65834896 Email: info@pepmic.com Web: www.pepmic.com
Address: 12 Datong Road, High-tech Development Zone, Suzhou, China 215151

pepmic
COMMITTED TO LIFE SCIENCE

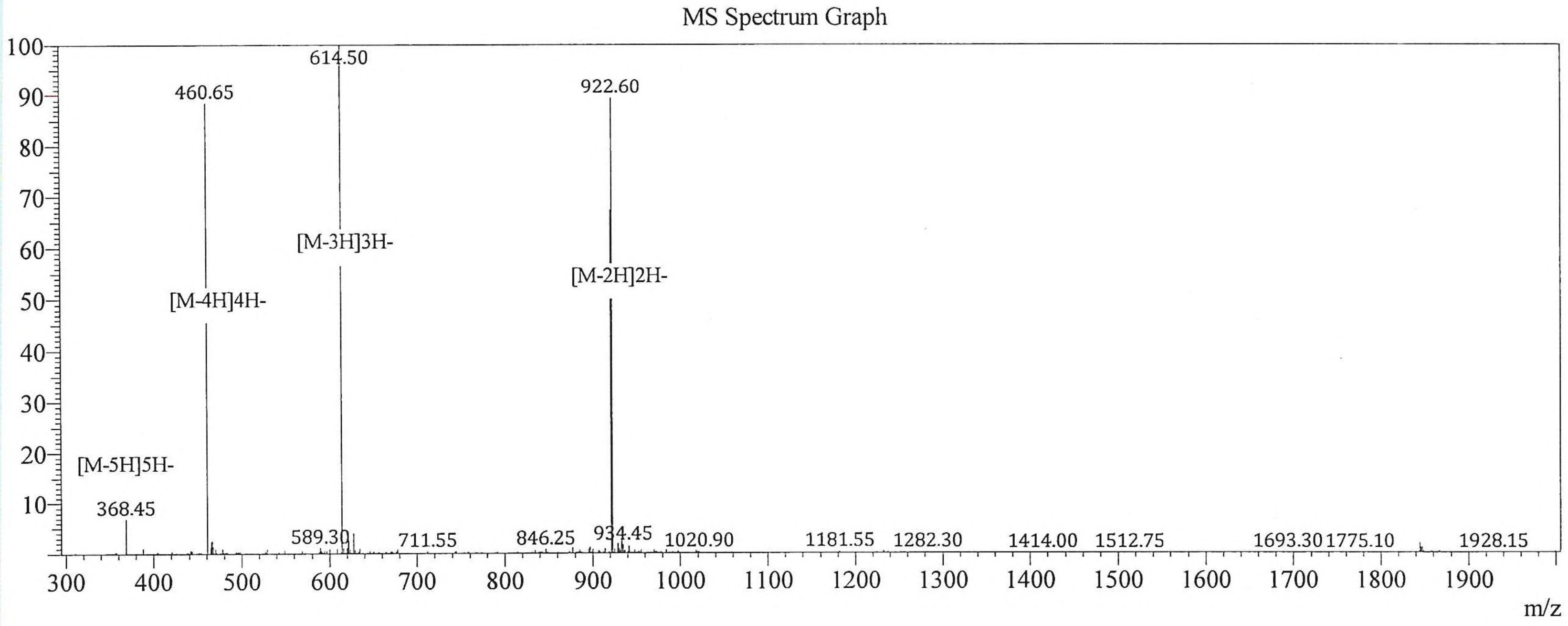

Sample Information

Dissolution method	:5%NH3.H2O+8%ACN+87%H2O	Interface	:ESI	Prerod Bias	:+1.5kv
Modified Date	:2022/10/10	Nebulizing Gas Flow	:1.50L/min	Detector	:-0.2kv
Injection Volume	:1ul	CDL Temp	:250C	T.Flow	:0.2ml/min
Heat Block Temp	:200	CDL Volt	:0v	B.conc	:50%H2O/50%MEOH

Name	:Pep6
Sequence	:Ac-D{E(Bn)}{t(Bn)}Ef(pt)(pY)LEc*Dc*-NH2(desalted)
Lot.No	:PCM15546-3-0919
Theoretical	:1846.65
Observed	:1846.50
